# Artificial intelligence in radiology: 173 commercially available products and their scientific evidence

**DOI:** 10.1007/s00330-025-11830-8

**Published:** 2025-07-24

**Authors:** Noa Antonissen, Olga Tryfonos, Ignas B. Houben, Colin Jacobs, Maarten de Rooij, Kicky G. van Leeuwen

**Affiliations:** 1 Department of Medical Imaging, Radboud University Medical Center, Nijmegen, The Netherlands; 2 Department of Dentistry, School of Medicine, European University Cyprus, Nicosia, Cyprus; 3 Department of Medical Imaging, Isala, The Netherlands; 4 Romion Health, Utrecht, The Netherlands; 5 Health AI Register, Utrecht, The Netherlands

**Keywords:** Artificial intelligence, Radiology, Device approval, Evidence-based practice

## Abstract

**Objectives:**

To assess changes in peer-reviewed evidence on commercially available radiological artificial intelligence (AI) products from 2020 to 2023, as a follow-up to a 2020 review of 100 products.

**Materials and methods:**

A literature review was conducted, covering January 2015 to March 2023, focusing on CE-certified radiological AI products listed on www.healthairegister.com. Papers were categorised using the hierarchical model of efficacy: technical/diagnostic accuracy (levels 1–2), clinical decision-making and patient outcomes (levels 3–5), or socio-economic impact (level 6). Study features such as design, vendor independence, and multicentre/multinational data usage were also examined.

**Results:**

By 2023, 173 CE-certified AI products from 90 vendors were identified, compared to 100 products in 2020. Products with peer-reviewed evidence increased from 36% to 66%, supported by 639 papers (up from 237). Diagnostic accuracy studies (level 2) remained predominant, though their share decreased from 65% to 57%. Studies addressing higher-efficacy levels (3–6) remained constant at 22% and 24%, with the number of products supported by such evidence increasing from 18% to 31%. Multicentre studies rose from 30% to 41% (*p* < 0.01). However, vendor-independent studies decreased (49% to 45%), as did multinational studies (15% to 11%) and prospective designs (19% to 16%), all with *p* > 0.05.

**Conclusion:**

The increase in peer-reviewed evidence and higher levels of evidence per product indicate maturation in the radiological AI market. However, the continued focus on lower-efficacy studies and reductions in vendor independence, multinational data, and prospective designs highlight persistent challenges in establishing unbiased, real-world evidence.

**Key Points:**

***Question*** *Evaluating advancements in peer-reviewed evidence for CE-certified radiological AI products is crucial to understand their clinical adoption and impact*.

***Findings*** *CE-certified AI products with peer-reviewed evidence increased from 36% in 2020 to 66% in 2023, but the proportion of higher-level evidence papers (~24%) remained unchanged*.

***Clinical relevance*** *The study highlights increased validation of radiological AI products but underscores a continued lack of evidence on their clinical and socio-economic impact, which may limit these tools’ safe and effective implementation into clinical workflows*.

**Graphical Abstract:**

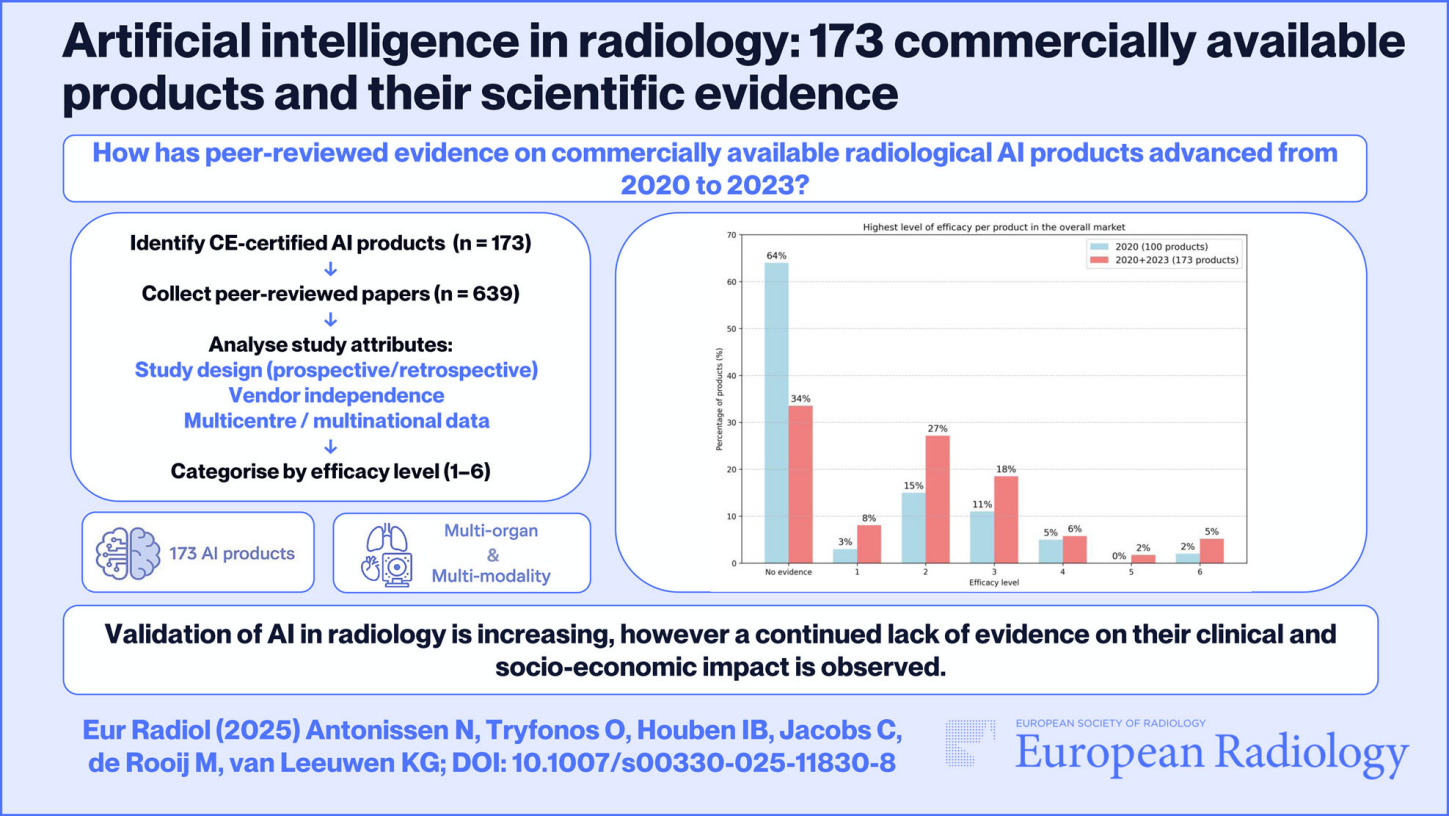

## Introduction

Artificial intelligence (AI) holds significant promise for improving diagnostic accuracy and streamlining clinical workflows in radiology [1, 2]. Despite the availability of numerous AI solutions [3], their widespread adoption remains limited. Key barriers include concerns about data privacy, difficulties integrating AI systems into existing workflows, unclear return on investment, and, most critically, uncertainties regarding their clinical value [4–7].

The Medical Device Regulation (MDR) requires each AI product to provide clinical evidence and undergo prospective monitoring as a prerequisite for Conformité Européenne/European Conformity (CE) certification in the European Union [8]; however, this data is not publicly available. As a result, peer-reviewed papers are often the only accessible source for evaluating the efficacy and real-world performance of these products, making them essential for guiding evidence-based adoption in radiology.

By 2020, only 36 out of 100 CE-certified radiological AI products had supporting evidence published in peer-reviewed literature [9]. Most of these studies had focused on technical performance and diagnostic accuracy, with minimal investigation into broader outcomes such as impact on clinical decision-making and patient care, or socio-economic benefits. This lack of comprehensive evidence raises critical questions about the readiness of AI technologies to meet the demands of real-world clinical practice.

This study systematically evaluated the evolution of peer-reviewed evidence for CE-certified radiological AI products between 2020 and 2023. Using an adapted hierarchical model of efficacy [9, 10], we assess advancements in demonstrating clinical relevance and patient-centred outcomes. Additionally, we examine trends such as multicentre collaborations, data diversity, and the independence of studies. Our goal is to provide insights into the current maturity of AI in radiology, identify remaining evidence gaps, and propose strategies to accelerate AI adoption while enhancing its clinical impact.

## Materials and methods

### Product selection

This study builds upon the 2020 analysis conducted by van Leeuwen et al [9], which assessed 100 radiological AI products and their associated peer-reviewed literature published between 1 January 2015 and 18 May 2020. This update (hereafter referred to as the 2023 analysis) focuses on CE-certified radiological AI products listed on www.healthairegister.com as of 31 March 2023, along with peer-reviewed papers published between 1 January 2015 and 31 March 2023. The analysis exclusively includes vendor-neutral imaging analysis tools developed using machine learning or deep learning techniques. Vendor and product names were revised to reflect the latest updates as of March 2023.

Excluded from the study were products discontinued before March 2023, vendors offering multi-task software suites, and tools focused on cardiac ultrasound or semi-automated cardiac segmentation. Discrepancies between analysed products and website listings may be due to recent updates to the website or the application of stricter inclusion criteria in this study.

### Scientific evidence selection

Scientific evidence was collected through a two-step approach. First, systematic searches on PubMed were conducted to identify peer-reviewed papers published between 1 January 2015 and 31 March 2023, using vendor and/or product names as search terms (queries provided in Supplementary Table S1). Second, additional peer-reviewed papers were obtained from www.healthairegister.com and the websites of respective vendors.

Eligible studies included prospective and retrospective original research articles in English that referenced the vendor or product name (including former names) in the methods or results section. Excluded were reviews, letters, editorials, commentaries, study protocols, case series, and case reports.

Three authors (N.A., O.T., and I.B.H.) independently screened titles, abstracts, and full texts against the inclusion criteria, followed by cross-checks and consensus meetings with two additional reviewers (M.d.R. and K.G.v.L.). Disagreements were resolved collaboratively.

### Analysis

Following the methodology outlined by van Leeuwen et al [9], studies were categorised based on the hierarchical model of efficacy, spanning from technical and diagnostic accuracy (levels 1 and 2) to clinical decision-making and patient outcomes (levels 3–5), and socio-economic implications (level 6). Definitions for these levels are detailed in Table 1. We present the available scientific evidence and the efficacy levels addressed in the papers, noting that a single paper could assess multiple levels.

**Table 1:** Hierarchical model of efficacy to assess the contribution of AI software to the diagnostic imaging process, adapted from Fryback and Thornbury (1991)

Level	Explanation	Typical measures
Level 1t	Technical efficacy: article demonstrates the technical feasibility of the software.	Reproducibility, inter-software agreement, error rate.
Level 1c	Potential clinical efficacy: article demonstrates the feasibility of the software to be clinically applied.	Correlation to alternative methods, potential predictive value, and biomarker studies.
Level 2	Diagnostic accuracy efficacy: article demonstrates the stand-alone performance of the software.	Standalone sensitivity, specificity, area under the ROC curve, or Dice score.
Level 3	Diagnostic thinking efficacy: article demonstrates the added value to the diagnosis.	Radiologist performance with/without AI, change in radiological judgement.
Level 4	Therapeutic efficacy: article demonstrates the impact of the software on the patient management decisions.	Effect on treatment or follow-up examinations.
Level 5	Patient outcome efficacy: article demonstrates the impact of the software on patient outcomes.	Effect on quality of life, morbidity or survival.
Level 6	Societal efficacy: article demonstrates the impact of the software on society by performing an economic analysis.	Effect on costs and quality-adjusted life years, incremental costs per quality-adjusted life year.

*Level 1t* level 1 (technical), *Level 1c* Level 1 (clinical)

Each article was reviewed for its author list, funding sources, and disclosures to determine whether it was vendor-independent. The data used in the included studies were categorised by the number of centres, countries, and imaging machine manufacturers from which it originated.

Additionally, a sub-analysis was performed on products featured in both the 2020 and 2023 analyses to examine market trends for products with longer market presence, which were expected to show greater maturation. This was contrasted with the broader market of 173 products, a significant portion of which were introduced after May 2020.

Statistical analysis involved summarising categorical variables as counts and percentages. Percentage changes between 2020 and 2023 were calculated, including 95% confidence intervals (CIs) derived using standard error propagation and the *Z*-score for a 95% confidence level. Proportions were compared using the chi-square test when all expected frequencies were ≥ 5, while Fisher’s exact test was applied for smaller expected frequencies. Statistical analyses were conducted in R (version 4.3.1); *p*-values less than 0.05 were considered statistically significant.

## Results

### Product overview and market trends

A total of 173 CE-certified radiological AI products from 90 vendors were included in this analysis. Of these, 85 were present in both the 2020 and 2023 analyses. The 15 products from the 2020 dataset that were excluded in 2023 consisted of 13 removed due to discontinuation or their absence from www.healthairegister.com, and 6 smaller products that were consolidated into 4 expanded-feature products. Additionally, 88 newly listed products since 2020 met the inclusion criteria, bringing the total number of CE-certified AI products evaluated in 2023 to 173.

**Fig. 1:**
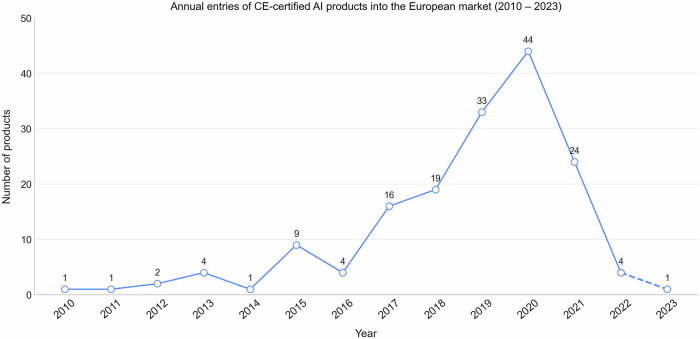
Yearly count of CE-certified radiological AI products introduced to the European market from 2010 to 2023. Products introduced before 2010 and those with unknown entry dates (*n* = 5 each) were excluded. The dashed line indicates that data for 2023 are incomplete, reflecting entries only up to 31 March 2023

**Fig. 2:**
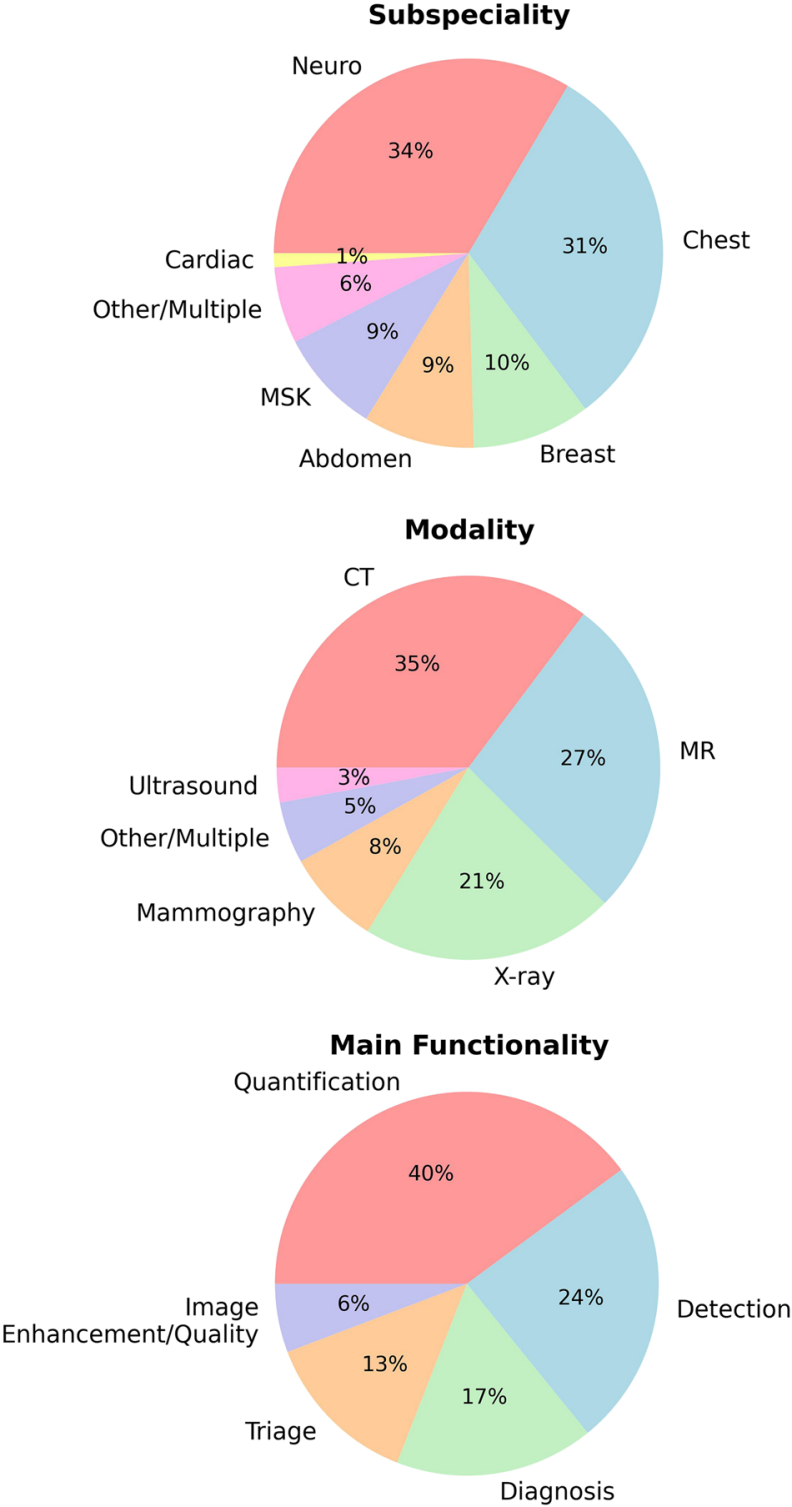
Characteristics of 173 CE-marked AI products categorised by organ-based subspeciality, modality, and main functionality. MSK, musculoskeletal

Figure 1 illustrates the annual number of new CE-certified radiological AI products entering the European market from 2010 to 2023. The data show a steady increase in new products over the years, with a peak of 44 in 2020, compared to 33 in 2019. This was followed by a decline, with 24 new products introduced in 2021 and only 4 in 2022. A detailed list of the products and vendors analysed is provided in Supplemental Table S2, while Fig. 2 shows their distribution across organ-based subspecialities, modalities, and functionalities.

### Trends in available peer-reviewed evidence and study characteristics

**Fig. 3:**
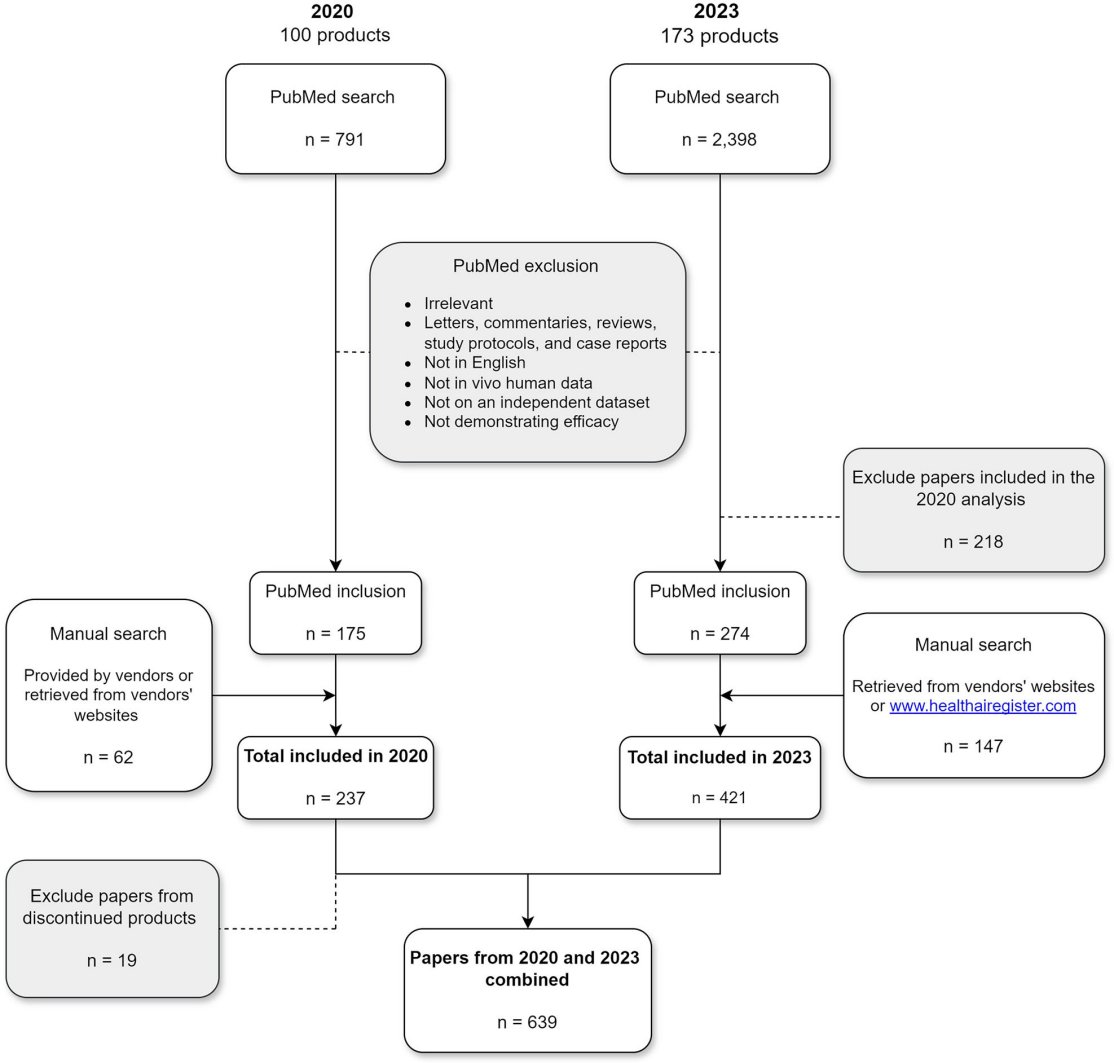
Flowchart summarising the inclusion of peer-reviewed evidence in the 2020 and 2023 analyses, and the final combined dataset

The PubMed search strategies identified a total of 2398 papers, of which 274 met the inclusion criteria and were not part of the 2020 analysis. An additional 147 papers were identified through manual searches, bringing the total number of included peer-reviewed articles to 421. Detailed search queries and their results are provided in Table S1 of the supplementary materials. Including papers from the 2020 analysis brought the total to 639 papers, after excluding 19 studies related to discontinued products. Articles could be listed multiple times in the analysis if they addressed more than one AI product, resulting in 78 entries derived from 36 unique papers. The inclusion process is summarised in the flowchart presented in Fig. 3.

**Fig. 4:**
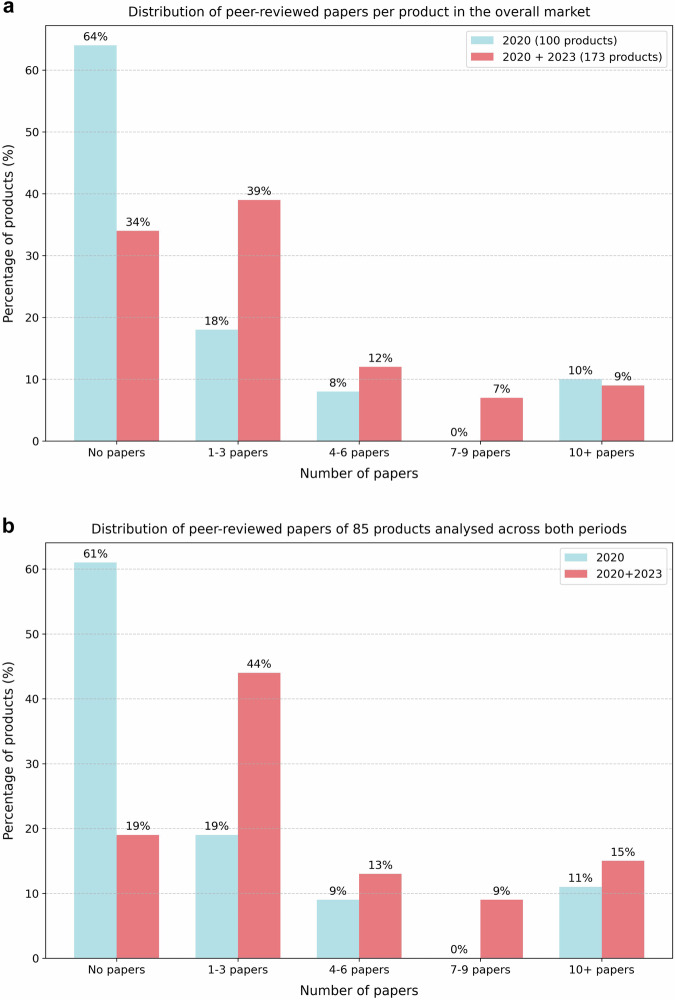
Distribution of peer-reviewed papers per product in (**a**) the overall market, analysing 100 products in 2020 and an expanded set of 173 products in 2023, and (**b**) a subset including 85 products that were analysed across both the 2020 and 2023 analyses

This update revealed a significant rise in peer-reviewed evidence for the AI products analysed. By 2023, 66% of the 173 CE-certified AI products included in the analysis were supported by peer-reviewed papers, compared to 36% of the 100 products assessed in 2020 (Fig. 4a). Among the 85 products evaluated in both 2020 and 2023, the proportion of products with evidence doubled, increasing from 39% to 81% (Fig. 4b). The number of products with 1–3 papers available doubled, rising from 18% to 39% across the overall market and from 19% to 44% within the subset of 85 continuously assessed products.

The changes in study characteristics between the papers assessed in the 2020 and 2023 analyses are summarised in Table 2. Studies using multicentre data increased significantly (30% to 41%, *p* < 0.01), as did the use of data from multiple manufacturers (25% to 34%, *p* = 0.02). However, vendor-independent studies decreased slightly (49% to 45%, *p* = 0.29), as did multinational studies (15% to 11%, *p* = 0.10) and prospective designs (19% to 16%, *p* = 0.28).

**Table 2:** Comparative analysis of research study characteristics across the 2020 and 2023 analyses

Metric	2020	2023	% Difference [95% CI]	*p*-value*
Total studies	237 (100%)	421 (100%)	N/A	N/A
Independent of the vendor	116 (48.9%)	188 (44.7%)	−4.29 [−12.23, 3.65]	0.29
Prospective design	45 (19.0%)	66 (15.7%)	−3.31 [−9.39, 2.77]	0.28
Multicentre data	71 (30.0%)	172 (40.9%)	10.90 [3.41, 18.38]	< 0.01
Multinational data	35 (14.8%)	44 (10.5%)	−4.32 [−9.70, 1.06]	0.10
Data acquired from multiple manufacturers	59 (24.9%)	142 (33.7%)	8.83 [1.71, 15.96]	0.02

*N/A* not applicable

* *p*-values are based on chi-square tests without continuity correction

### Trends in scientific evidence by levels of efficacy

**Fig. 5:**
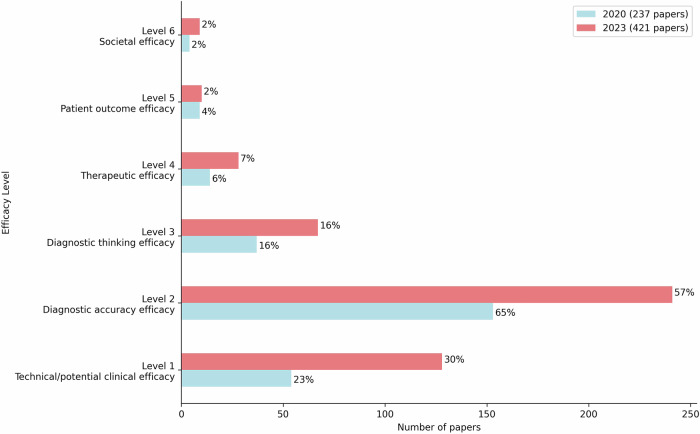
Levels of efficacy evaluated in peer-reviewed papers included in the 2020 and 2023 analyses. The values next to each bar represent the percentage of papers at each efficacy level, relative to the total number of papers analysed in that year (2020: *n* = 237; 2023: *n* = 421). A single paper could address multiple levels of efficacy; therefore, the percentages do not add up to 100%

The trends in efficacy levels assessed in peer-reviewed papers supporting AI products from the 2020 and 2023 analyses are shown in Fig. 5 and Supplemental Table S3. Studies assessing technical or clinical feasibility (level 1) saw a significant increase (*p* = 0.04), rising from 23% (54/237) in 2020 to 30% (128/421) in 2023. In contrast, no statistically significant changes were observed in studies at other efficacy levels. Diagnostic accuracy (level 2) remained the predominant focus, though its share declined from 65% (153/237) in 2020 to 57% (241/421) in 2023. Meanwhile, research addressing higher-efficacy levels (levels 3–6), which evaluate clinical and socio-economic impacts, grew in absolute terms from 53 studies in 2020 to 99 in 2023, though their relative share remained steady at ±24%. To better understand where this evidence is concentrated, Supplementary Fig. S1 illustrates the distribution of low- and high-level evidence peer-reviewed papers by subspeciality, imaging modality, and functionality, showing that most studies remain concentrated in chest and neuroimaging, with CT and X-ray as the dominant modalities, and quantification and diagnosis as the leading functionalities.

**Fig. 6:**
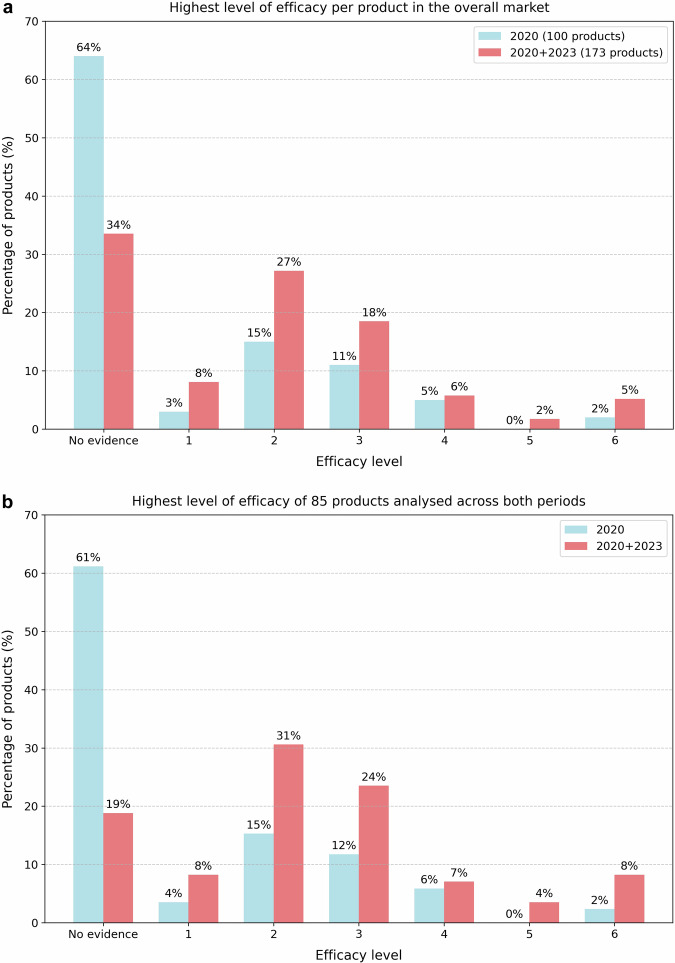
Highest level of efficacy applicable per product in (**a)** the overall market, including 100 products in the 2020 analysis and 173 products in the 2023 analysis, and (**b**) a subset including 85 products that were analysed across both the 2020 and 2023 analyses

When considering the highest level of efficacy applicable per product, Fig. 6a shows that 64% of the 100 products analysed in 2020 lacked any efficacy evidence. By 2023, this proportion dropped to 34% among the 173 products analysed. Products with evidence demonstrating technical or diagnostic accuracy (levels 1 and 2) increased from 18% to 35%, while those with evidence addressing clinical and socio-economic impacts (levels 3–6) rose from 18% to 31%. Supplementary Fig. S2 further breaks down the distribution of products by subspeciality based on their highest level of supporting evidence. Neuroimaging accounted for the largest number of CE-marked AI products, though most lacked support or were backed only by lower-level evidence. In contrast, chest imaging had the highest number of products supported by higher-level efficacy evidence.

For the 85 products evaluated in both 2020 and 2023, the proportion without any efficacy evidence declined substantially, from 61% in 2020 to 19% in 2023, as shown in Fig. 6b. Among these products, evidence for technical or diagnostic accuracy (levels 1 and 2) rose from 19% to 39%, while evidence supporting clinical or socio-economic impacts (levels 3–6) increased from 20% to 43%.

## Discussion

This follow-up study evaluates the evolving scientific evidence supporting CE-certified radiological AI products, building on the 2020 analysis by van Leeuwen et al [9]. Since 2020, the market has shifted from rapid product expansion to a more mature state. New certifications peaked in 2020 but declined sharply thereafter, likely due to a combination of market saturation reducing investor interest [11] and the introduction of the MDR [8]. The MDR introduced more stringent requirements, including enhanced clinical evaluation standards, comprehensive post-market surveillance obligations, and more detailed technical documentation requirements, increasing the complexity, duration, and costs of the certification process.

In parallel with these regulatory and market shifts, peer-reviewed evidence supporting CE-certified radiological AI products has increased substantially. By 2023, 66% of the 173 products analysed in this study were supported by at least one peer-reviewed publication, up from 36% of 100 products in 2020. Products showing technical feasibility or diagnostic accuracy (levels 1 and 2) increased from 18% to 35%, while those addressing clinical or socio-economic outcomes (levels 3 to 6) grew from 18% to 31%. This trend was even more pronounced among the 85 products analysed in both time periods, reflecting the benefits of longer market presence and maturation.

However, the distribution of supporting evidence varies across radiological domains. Chest imaging products account for the largest number of tools with higher-level validation, likely due to the prevalence of thoracic diseases, the availability of standardised datasets, and well-established diagnostic tasks. In contrast, neuroimaging, which represents the largest category of CE-certified products, is more frequently supported by lower-level studies. Many neuro-AI tools focus on volumetric analysis or segmentation tasks, which are less suited to prospective, outcome-based evaluation. In addition, challenges such as data heterogeneity, limited integration into clinical workflows, and the lack of established clinical benchmarks may hinder the development of higher-level validation in neuroradiology [12].

Despite the overall growth in published evidence and the number of products supported by peer-reviewed studies, most publications continue to focus on lower levels of efficacy, with limited progress in evaluating clinical or socio-economic outcomes. Between 2020 and 2023, the proportion of studies addressing these higher-level impacts remained stable at approximately 24%. This stagnation suggests that while the volume of evidence has increased, its clinical relevance has not kept pace, limiting the ability of healthcare providers and policymakers to assess the real-world value of these tools.

These findings align with observations by Chouffani et al [13] and Brady et al [14], who note that regulatory approval does not guarantee clinical effectiveness, especially when validations rely heavily on retrospective studies. They stress the importance of independent evaluations to reduce vendor bias and ensure real-world utility and safety. Moreover, reliance on single-site studies and dataset biases limits generalisability and raises ethical concerns about transparency and equity in outcomes across diverse patient populations [15, 16]. This lack of generalisability is further exacerbated by insufficient consideration of variations in clinical workflows, patient demographics, and healthcare infrastructure, making it difficult to apply findings across diverse settings. Addressing these challenges requires diverse, representative datasets and continuous learning frameworks to ensure adaptability and sustained performance in dynamic clinical environments [17, 18]. These frameworks enable AI systems to evolve with changing data and settings, enhancing robustness while addressing transparency and equity concerns. Without these measures, AI integration into clinical practice risks being undermined by limited reliability, applicability, and trustworthiness.

This study has several limitations that reflect the challenges of evaluating AI tools in a rapidly evolving landscape. The data, limited to March 2023, may already be outdated as new products and validation studies continue to emerge. Notable examples include the MASAI and PRAIM trials, which represent high-level real-world evidence for breast cancer screening AI and were published after our inclusion period [19, 20]. While these trials signal progress towards more rigorous clinical validation, such prospective evidence remains largely concentrated in breast imaging and is not yet widely observed across other domains. Additionally, the aggregation of findings across diverse radiological domains offers a broad perspective but may obscure domain-specific differences in evidence maturity and clinical applicability. To mitigate this, the supplementary materials include targeted sub-analyses that highlight the heterogeneity across domains. Finally, the definition of AI in the context of clinical radiology is complex and not easily standardised, making our product inclusion criteria a potential source of debate. For example, cardiac ultrasound tools were excluded due to their frequent management within cardiology rather than radiology departments. Despite these limitations, we believe this study provides important insights into the current state of AI in radiology, highlighting significant trends and ongoing challenges in the field.

In conclusion, this study underscores both progress and persistent challenges in the validation of CE-certified radiological AI products. The growing proportion of products with peer-reviewed evidence reflects a positive trend, yet the concentration of studies at lower efficacy levels highlights a critical gap in demonstrating clinical impact and improved patient outcomes. While the increasing maturity of products and market presence suggest a movement towards higher-level validations, the stagnation in studies addressing clinical and socio-economic efficacy points to the need for more rigorous, real-world assessments.

### Future perspective

To address these gaps, we believe future efforts should focus on improving the quality and scope of validation studies, with an emphasis on diverse, multicentre, prospective validations that reflect real-world clinical settings, patient populations, and healthcare systems. Funding agencies have a crucial role to play by prioritising calls specifically aimed at supporting not only the development but also the independent validation, implementation, and long-term monitoring of AI tools in clinical practice. Under the MDR [8], vendors are already required to monitor the clinical performance of their solutions through post-market surveillance. Although publishing these findings is not mandatory, sharing such data could provide valuable insights into the real-world effectiveness of AI tools. We encourage both AI solution developers and users to contribute to this collective knowledge to strengthen transparency, reliability, and support for healthcare providers, ultimately advancing the safe and effective adoption of AI into clinical workflows.

## Supplementary information

ELECTRONIC SUPPLEMENTARY MATERIAL

## Abbreviations

AI: Artificial intelligence
CE: Conformité Européenne/European Conformity
CI: Confidence interval
MDR: Medical device regulation
