# Supplementary material for: Artificial intelligence in radiology: 173 commercially available products and their scientific evidence
Source: Eur Radiol. 2025 Jul 24;36(1):526–36. doi: 10.1007/s00330-025-11830-8 (PMC12711992; doi:10.1007/s00330-025-11830-8)
Supplement: Supplementary file 1 — ELECTRONIC SUPPLEMENTARY MATERIAL [file 330_2025_11830_MOESM1_ESM.pdf]

Artificial intelligence in radiology: 173 commercially available products and their scientific evidence

ELECTRONIC SUPPLEMENTARY MATERIAL

Vendor	Query PubMed	Hits PubMed	Included PubMed*	Included manual*	Included total*
Advantis	(("2015"[Date - Publication] : "3000"[Date - Publication])) AND (advantis OR brainance)	8	2	0	2
*Aidence***	(("2015"[Date - Publication] : "3000"[Date - Publication])) AND (aidence OR veye chest OR veye lung nodules)	31	3	2	5
Aidoc	(("2015"[Date - Publication] : "3000"[Date - Publication])) AND (aidoc)	16	6	11	17
AIRAmed	(("2015"[Date - Publication] : "3000"[Date - Publication])) AND (airamed OR airascore)	13	2	0	2
AlgoMedica	(("2015"[Date - Publication] : "3000"[Date - Publication])) AND (algomedica OR pixelshine)	12	9	1	10
AmCad BioMed	(("2015"[Date - Publication] : "3000"[Date - Publication])) AND (amcad)	12	5	0	5
annalise.ai	(("2015"[Date - Publication] : "3000"[Date - Publication])) AND (annalise)	29	5	0	5
Avicenna.AI	(("2015"[Date - Publication] : "3000"[Date - Publication])) AND (avicenna.ai)	11	3	2	5
AZmed	(("2015"[Date - Publication] : "3000"[Date - Publication])) AND (AZmed AND fracture) OR (rayvolve)	1	1	2	3
Behold.ai	(("2015"[Date - Publication] : "3000"[Date - Publication])) AND (behold.ai OR red dot algorithm)	19	2	0	2
Brainminer	(("2015"[Date - Publication] : "3000"[Date - Publication])) AND (brainminer)	9	0	0	0
Brainomix	(("2015"[Date - Publication] : "3000"[Date - Publication])) AND (brainomix AND (e-aspects OR e-stroke OR e-CTA OR e-CTP))	23	13	1	14
Brainreader	(("2015"[Date - Publication] : "3000"[Date - Publication])) AND (brainreader OR neuroreader)	21	15	0	15
BrainScan Ltd	(("2015"[Date - Publication] : "3000"[Date - Publication])) AND (brainscan.ai OR brainscan)	37	0	0	0
BrainTale	(("2015"[Date - Publication] : "3000"[Date - Publication])) AND (braintale)	13	1	0	1
b-rayZ	(("2015"[Date - Publication] : "3000"[Date - Publication])) AND ((b-rayz OR b rayz OR rayz OR b-box) AND breast)	3	0	0	0
Cercare Medical	(("2015"[Date - Publication] : "3000"[Date - Publication])) AND (cercare)	5	0	0	0
Cerebriu	(("2015"[Date - Publication] : "3000"[Date - Publication])) AND (cerebriu)	11	0	0	0
Circle Neurovascular Imaging	(("2015"[Date - Publication] : "3000"[Date - Publication])) AND ((circle AND "neurovascular imaging") OR strokesens)	3	2	0	2
Combinostics	(("2015"[Date - Publication] : "3000"[Date - Publication])) AND (combinostics AND cneuro)	2	2	1	3

Contextflow	(("2015"[Date - Publication] : "3000"[Date - Publication])) AND (contextflow)	13	1	0	1
Coreline Soft	(("2015"[Date - Publication] : "3000"[Date - Publication])) AND ("coreline soft" OR aview)	53	10	12	22
Cortechs.ai	(("2015"[Date - Publication] : "3000"[Date - Publication])) AND (neuroquant)	50	10	2	12
Deep01 Limited	(("2015"[Date - Publication] : "3000"[Date - Publication])) AND (deepCT)	4	1	1	2
DeepTrace Technologies Srl	(("2015"[Date - Publication] : "3000"[Date - Publication])) AND (deeptrace OR trace4AD OR trace4OC)	20	0	0	0
Delft Imaging	(("2015"[Date - Publication] : "3000"[Date - Publication])) AND ((CAD4TB OR CAD4COVID)	33	15	0	15
Densitas	(("2015"[Date - Publication] : "3000"[Date - Publication])) AND (densitas OR intellimammo OR densitasai)	3	2	0	2
Digitec	(("2015"[Date - Publication] : "3000"[Date - Publication])) AND (digitec AND tiresya)	8	0	0	0
GE Healthcare	(("2015"[Date - Publication] : "3000"[Date - Publication])) AND ((GE AND Healthcare) AND (Critical AND Care AND Suite))	19	0	0	0
Gleamer	(("2015"[Date - Publication] : "3000"[Date - Publication])) AND (Gleamer OR BoneView OR ChestView)	12	5	3	8
Hanalytics (BioMind)	(("2015"[Date - Publication] : "3000"[Date - Publication])) AND ((hanalytics OR biomind))	35	3	0	3
HeartFlow	(("2015"[Date - Publication] : "3000"[Date - Publication])) AND (Heartflow AND FFRCT)	90	15	4	19
Hera-MI	(("2015"[Date - Publication] : "3000"[Date - Publication])) AND (Hera-MI OR Slimview)	3	0	0	0
iCAD	(("2015"[Date - Publication] : "3000"[Date - Publication])) AND (iCAD AND (profound OR "breast cancer"))	41	5	2	7
icometrix	(("2015"[Date - Publication] : "3000"[Date - Publication])) AND (icometrix OR icobrain OR icolung OR msmetrix)	153	6	0	6
ImageBiopsy Lab	(("2015"[Date - Publication] : "3000"[Date - Publication])) AND ("Image Biopsy Lab" OR "IB Lab KOALA" OR "IB Lab PANDA" OR "IB Lab HIPPO" OR "IB Lab LAMA")	12	6	2	8
Imaging Biometrics	(("2015"[Date - Publication] : "3000"[Date - Publication])) AND ("Imaging Biometrics" OR "StoneChecker")	22	7	3	10
Imbio	(("2015"[Date - Publication] : "3000"[Date - Publication])) AND (Imbio AND Lung)	66	4	0	4
IMedis	(("2015"[Date - Publication] : "3000"[Date - Publication])) AND (IMedis OR DualiQ)	15	0	0	0
Incepto	(("2015"[Date - Publication] : "3000"[Date - Publication])) AND (Incepto)	8	3	0	3
Infervision	(("2015"[Date - Publication] : "3000"[Date - Publication])) AND (Infervision OR Inferread) AND (Tuberculosis OR Chest OR Pneumonia OR Lung)	47	9	5	14
JLK Inc.	(("2015"[Date - Publication] : "3000"[Date - Publication])) AND (JLK) AND (Breast OR Chest OR Neuro OR Abdomen) AND ("artificial intelligence")	9	0	0	0
Kheiron Medical Technologies	(("2015"[Date - Publication] : "3000"[Date - Publication])) AND ("Kheiron Medical Technologies")	21	1	0	1
Koios Medical, Inc.	(("2015"[Date - Publication] : "3000"[Date - Publication])) AND (Koios)	11	2	3	5
Lucida Medical	(("2015"[Date - Publication] : "3000"[Date - Publication])) AND (Lucida AND "Prostate Intelligence")	1	0	0	0

Lunit	(("2015"[Date - Publication] : "3000"[Date - Publication])) AND (Lunit AND Insight)	21	18	11	29
Mediaire	(("2015"[Date - Publication] : "3000"[Date - Publication])) AND (Mediaire OR Mdbrain)	11	1	3	4
Medimaps Group	(("2015"[Date - Publication] : "3000"[Date - Publication])) AND (Medimaps AND "TBS iNsight")	20	0	15	15
MeVis Medical Solutions AG	(("2015"[Date - Publication] : "3000"[Date - Publication])) AND ("MeVis Medical Solutions" AND Veolity)	7	2	0	2
Milvue	(("2015"[Date - Publication] : "3000"[Date - Publication])) AND (Milvue OR SmartUrgences)	3	2	1	3
Monitor Corporation	(("2015"[Date - Publication] : "3000"[Date - Publication])) AND ("Monitor Corporation" AND LuCAS)	12	0	0	0
Motilent	(("2015"[Date - Publication] : "3000"[Date - Publication])) AND (Motilent OR GIQuant)	17	0	5	5
MRIguidance	(("2015"[Date - Publication] : "3000"[Date - Publication])) AND (MRIguidance AND Bone)	14	1	7	8
Nanox.AI	(("2015"[Date - Publication] : "3000"[Date - Publication])) AND (Nanox OR HealthCCSng OR "Zebra medical vision")	43	0	1	1
Neurophet	(("2015"[Date - Publication] : "3000"[Date - Publication])) AND (Neurophet)	14	0	0	0
Nicolab	(("2015"[Date - Publication] : "3000"[Date - Publication])) AND (Nico-Lab OR Nico.lab OR StrokeViewer)	77	4	0	4
Olea Medical	(("2015"[Date - Publication] : "3000"[Date - Publication])) AND ("Olea Medical" OR breastscape) AND (Breast)	24	1	0	1
Optellum	(("2015"[Date - Publication] : "3000"[Date - Publication])) AND (Optellum)	42	7	1	8
Oxipit	(("2015"[Date - Publication] : "3000"[Date - Publication])) AND (Oxipit OR ChestLink OR ChestEye)	3	1	1	2
Paire	(("2015"[Date - Publication] : "3000"[Date - Publication])) AND (PAIRE OR Pionus)	21	0	0	0
Perspectum	(("2015"[Date - Publication] : "3000"[Date - Publication])) AND (Perspectum) AND (MRCP+ OR LiverMultiScan)	37	4	1	5
Pixyl	(("2015"[Date - Publication] : "3000"[Date - Publication])) AND (Pixyl)	6	2	0	2
*Quantib***	(("2015"[Date - Publication] : "3000"[Date - Publication])) AND (Quantib)	75	5	1	6
Qubiotech	(("2015"[Date - Publication] : "3000"[Date - Publication])) AND (Qubiotech OR Neurocloud)	5	0	0	0
Quibim	(("2015"[Date - Publication] : "3000"[Date - Publication])) AND (Quibim)	53	2	2	4
Qure.ai	(("2015"[Date - Publication] : "3000"[Date - Publication])) AND (Qure.ai OR qXR OR qER)	41	9	2	11
QView Medical	(("2015"[Date - Publication] : "3000"[Date - Publication])) AND ("Qview Medical" OR QVCAD)	4	1	1	2
Qynapse	(("2015"[Date - Publication] : "3000"[Date - Publication])) AND (Qynapse OR QyScore)	57	1	1	2
Radiobotics	(("2015"[Date - Publication] : "2023"[Date - Publication])) AND (radiobotics OR rbknee OR rbfracture)	3	1	0	1
RapidAI	(("2015"[Date - Publication] : "3000"[Date - Publication])) AND (RapidAI)	12	4	6	10
Rayscape	(("2015"[Date - Publication] : "3000"[Date - Publication])) AND (Rayscape) AND (lung OR Xvision)	1	1	0	1

Resonance Health	(("2015"[Date - Publication] : "3000"[Date - Publication])) AND ("Resonance Health" OR FerriSmart OR "HepaFat-Scan")	32	2	2	4
RevealDx	(("2015"[Date - Publication] : "3000"[Date - Publication])) AND (RevealDx OR RevealAI OR "Mindshare Medical")	6	2	0	2
Riverain Technologies	("2015"[Date - Publication] : "3000"[Date - Publication])) AND (Riverain OR ClearRead)	55	3	0	3
Samsung Healthcare	(("2015"[Date - Publication] : "3000"[Date - Publication])) AND ("Samsung Electronics") AND (chest OR lung)	25	1	0	1
ScreenPoint Medical	(("2015"[Date - Publication] : "3000"[Date - Publication])) AND (“ScreenPoint Medical” OR Transpara)	41	1	10	11
SenseTime	(("2015"[Date - Publication] : "3000"[Date - Publication])) AND (SenseTime) AND (SenseCare)	0	0	0	0
Siemens Healthineers	(("2015"[Date - Publication] : "3000"[Date - Publication])) AND (Siemens) AND ("AI-Rad Companion" OR "AI Rad Companion")	10	4	8	12
Smart Soft Healthcare	(("2015"[Date - Publication] : "3000"[Date - Publication])) AND (”Smart Soft Healthcare”) AND (spine)	2	0	2	2
SyntheticMR	(("2015"[Date - Publication] : "3000"[Date - Publication])) AND (SyntheticMR AND neuro) OR ("SyMRI neuro")	25	2	1	3
Therapixel	(("2015"[Date - Publication] : "3000"[Date - Publication])) AND (Therapixel)	10	1	0	1
Thirona	(("2015"[Date - Publication] : "3000"[Date - Publication])) AND (Thirona) AND (lung OR CAD4COVID-CT)	65	2	2	4
Vara (MX Healthcare GmbH)	(("2015"[Date - Publication] : "3000"[Date - Publication])) AND ((Vara) AND (breast))	15	0	2	2
VIDA	(("2015"[Date - Publication] : "3000"[Date - Publication])) AND ("VIDA insights") AND (lung)	16	0	0	0
Visage Imaging, Inc.	(("2015"[Date - Publication] : "3000"[Date - Publication])) AND ((Visage) AND ("Breast Density"))	0	0	0	0
Visiana	("2015"[Date - Publication] : "3000"[Date - Publication])) AND (Visiana OR BoneXpert)	52	2	2	4
Visionairy Health	(("2015"[Date - Publication] : "3000"[Date - Publication])) AND ("Visionairy Health")	2	0	0	0
Viz.ai	(("2015"[Date - Publication] : "3000"[Date - Publication])) AND ("Viz.ai" OR "Viz LVO" OR "Viz CTP")	144	7	0	7
Volpara Health	(("2015"[Date - Publication] : "3000"[Date - Publication])) AND (Volpara OR VolparaDensity)	151	4	0	4
VUNO	(("2015"[Date - Publication] : "3000"[Date - Publication])) AND (Vuno OR BoneAge)	77	0	2	2

Table S1. Literature search queries, hits, and included studies per vendor.

* These numbers reflect only additional inclusions from the 2023 analysis and exclude papers from the 2020 analysis.

** Aidence and Quantib were renamed DeepHealth between the analysis and publication.

Vendor	Product	On the market since	In both 2020 and 2023 analysis	Name changes for vendors/products analysed in 2020
Advantis	Brainance MD	2018	No	
Aidoc	Brain aneurysm (BA)	2021	No	
	C-Spine (CSF)	2017	Yes	
	Incidental Pulmonary embolism (iPE)	2020	No	
	Intra-Abdominal Free Gas (IFG)	2020	No	
	Intracranial Hemorrhage (ICH)	2017	Yes	
	Ischemic Stroke	2019	Yes	Product name was Large Vessel Occlusion (LVO)
	Pneumothorax (Ptx)	2021	No	
	Pulmonary embolism (PE)	2019	Yes	
	Pulmonary nodules QC (PN QC)	2021	No	
	Rib fractures (RibFx)	2021	No	
AIRAmed	AIRAscore structure	2020	No	
AlgoMedica	PixelShine	2019	No	
AmCad BioMed	AmCAD-UT®	2017	Yes	
annalise.ai	Annalise Enterprise CXR	2020	No	
Avicenna.AI	CINA-AD	2020	No	
	CINA-ASPECTS	2021	No	
	CINA-ICH	2020	Yes	Product name was CINA ICH (without hyphen)
	CINA-LVO	2020	Yes	Product name was CINA LVO (without hyphen)
	CINA-PE	2020	No	
Azmed	Rayvolve Trauma	2019	Yes	Product name was Rayvolve
Behold.ai	red dot Chest X-Ray	2020	Yes	Product name was Red Dot
Brainminer	Diadem	?	Yes	

Brainomix	e-ASPECTS	2015	Yes	
	e-CTA	2018	Yes	
Brainreader	Neuroreader®	2015	No	
BrainScan Inc.	BrainScan CT	2020	No	
BrainTale	brainTale-care	2022	Yes	Product name was BrainQuant
b-rayZ	b-box plus	2020	No	
Cercare Medical	Cercare Perfusion	2019	No	
	Cercare Stroke	2019	No	
Cerebriu	Cerebriu Apollo Brain	2021	No	
Circle Neurovascular Imaging	StrokeSENS	2021	No	
Combinostics	cNeuro cMRI	2017	Yes	
contextflow	contextflow ADVANCE Chest CT	2019	No	
Coreline Soft	AVIEW CAC	2017	No	
	AVIEW COPD	2017	No	
	AVIEW LCS+	2017	No	
	AVIEW Lung Texture	2017	No	
Cortechs.ai	NeuroQuant	2006	Yes	
	NeuroQuant MS	2006	No	
Deep01 Limited	DeepCT	2019	No	
DeepHealth	Saige Brain (Quantib® ND)	2017	Yes	Vendor name was Quantib<br><br>Product name was Quantib® ND
	Saige Lung (Veye Lung Nodules)	2017	Yes	Vendor name was Aidence<br><br>Product name was Veye Chest
	Saige Prostate (QUANTIB® Prostate)	2020	No	
DeepTrace Technologies Srl	TRACE4AD	2021	No	
	TRACE4OC	2021	No	

Delft Imaging	CAD4COVID-Xray	2020	No	Vendor name was Thirona
	CAD4TB	2014	Yes	Vendor name was Thirona
Densitas	intelliMammo™	2017	Yes	Product name was densitasai
DiA Imaging Analysis	LVivo Toolbox – Abdominal	2020	No	
Digitec	TIRESYA	?	No	
GE Healthcare	Critical Care Suite	2019	No	
GLEAMER	BoneView	2020	Yes	Vendor name was Gleamer.ai
	ChestView	2021	No	
Hanalytics (BioMind)	Brain Tumours Application	2017	Yes	Product name was BioMind
HeartFlow	HeartFlow FFRCT Analysis	2016	Yes	
Hera-MI	Breast-SlimView	2020	Yes	
iCAD	ProFound AI for 2D Mammography	2019	Yes	
	ProFound AI for Digital Breast Tomosynthesis	2018	Yes	
	ProFound AI Risk	2020	No	
icometrix	icobrain cva	2020	No	
	icobrain dm	2018	Yes	
	icobrain ep	2020	Yes	
	icobrain ms	2016	Yes	
	icobrain tbi	2018	Yes	
	Icolung	2020	No	
ImageBiopsy Lab	IB Lab HIPPO	2020	No	
	IB Lab KOALA	2019	Yes	
	IB Lab LAMA	2020	No	
	IB Lab PANDA	2020	Yes	
Imaging Biometrics	IB DCE	2013	No	
	IB Delta Suite	2013	No	

	IB Diffusion	2013	No	
	IB Neuro	2018	No	
	StoneChecker	2019	No	
Imbio	Lung Density Analysis - Functional	2015	Yes	
	Lung Texture Analysis	2015	Yes	
Imedis	Duali-Q	?	No	
Incepto	ARVA	2021	No	
	KEROS	2021	No	
Infervision	InferRead CT Lung	2020	Yes	
	InferRead CT Pneumonia	2020	Yes	
	InferRead DR Chest	2020	Yes	
	InferRead DR Tuberculosis	2020	Yes	
JLK Inc.	JAD-02K	2019	Yes	
	JBA-01K	2019	Yes	
	JBD-01K	2020	Yes	
	JBS-01K	2019	Yes	
	JBS-04K	2019	Yes	
	JLD-01K	2019	Yes	
	JLD-02K	2019	Yes	
	JPC-01K	2019	Yes	
Kheiron Medical Technologies	Mia	2018	Yes	
Koios Medical, Inc.	Koios DS	2021	No	
Lucida Medical	Prostate Intelligence™	2021	No	
Lunit	Lunit INSIGHT CXR	2019	Yes	
	Lunit INSIGHT MMG	2020	No	
mediaire	Mdbrain	2019	Yes	
Medimaps Group	TBS iNsight (Osteo)	2012	No	
MeVis Medical Solutions AG	Veolity	2015	Yes	

Milvue	Milvue Suite - SmartUrgences	2020	No	
Monitor Corporation	LuCAS-Plus	2020	No	
Motilent	GIQuant®	2019	No	
MRIguidance	BoneMRI	2019	No	
Nanox.AI	HealthCCSng cardiac solution	2021	No	
Neurophet	Neurophet AQUA	2020	No	
Nicolab	StrokeViewer	2019	Yes	Vendor name was Nico.Lab<br><br>Previously analysed as two separate products: ‘Thrombus location’ and ‘Haemorrhage detection’
Olea Medical	breastscape® v1.0	2018	No	
Optellum	Virtual Nodule Clinic	?	No	
Oxipit	ChestEye Quality	2021	Yes	Product name was ChestEye CAD
	ChestLink	2022	No	
PAIRE	Pionus	2021	No	
Perspectum	LiverMultiScan	2017	No	
	MRCP+	2019	No	
Pixyl	Pixyl.Neuro.BV	2019	Yes	
	Pixyl.Neuro.FL	2019	Yes	
	Pixyl.Neuro.MS	2019	Yes	
Qubiotech	Neurocloud PET	2017	No	
	Neurocloud SISCOM	2018	No	
	Neurocloud VOL	2019	No	
Quibim	QP-Brain	2023	Yes	Multiple smaller products combined into one product
	QP-Liver	?	Yes	Multiple smaller products combined into one product
	QP-Prostate	2022	Yes	Multiple smaller products combined into one product

Qure.ai	qCT-Lung	2021	No	
	qER	2018	Yes	
	qXR	2018	Yes	
QView Medical	QVCAD	2016	Yes	
Qynapse	QyScore	2017	No	
Radiobotics	Rbfracture	2022	No	
	Rbknee	2019	Yes	
RapidAI	Rapid ASPECTS	2020	Yes	
	Rapid CTA	2018	Yes	
	Rapid CTP	2018	No	
	Rapid for Angio	2018	No	
	Rapid ICH	2020	Yes	
	Rapid MRI	2018	No	
Rayscape	Chest X-ray	2021	No	
	Lung CT	2021	No	
Resonance Health	FerriSmart	2018	Yes	
	HepaFat-Scan	2013	No	
RevealDx	RevealAI-Lung	2021	Yes	Vendor name was Mindshare Medical<br><br>Product name was RevealAI Lung (without hyphen)
Riverain Technologies	ClearRead CT – Compare	2015	Yes	
	ClearRead CT – Detect	2015	Yes	
	ClearRead CT - Vessel Suppress	2015	Yes	
	ClearRead Xray - Bone Suppress	2008	Yes	
	ClearRead Xray – Compare	2011	No	
	ClearRead XRay – Confirm	2012	Yes	
	ClearRead Xray – Detect	2008	Yes	
Samsung Healthcare	Auto Lung Nodule Detection	2018	No	

ScreenPoint Medical	Transpara	2015	Yes	
SenseTime	SenseCare-Chest DR Pro	2021	No	
	SenseCare-Lung Pro	2020	No	
Siemens Healthineers	AI-Rad Companion Brain MR	2020	Yes	
	AI-Rad Companion Chest CT	2019	Yes	
	AI-Rad Companion Chest X-ray	2020	Yes	
	AI-Rad Companion Prostate MR	2020	Yes	
	Prostate MR on syngo.via	2020	No	
Smart Soft Healthcare	CoLumbo	2021	No	
SyntheticMR	SyMRI NEURO	2017	Yes	
Therapixel	MammoScreen®	2020	No	
Thirona	LungQ™ Clinical Suite	2016	Yes	Product name was LungQ
Vara (MX Healthcare GmbH)	Vara	2019	Yes	Vendor name was Vara (Merantix Healthcare)
VIDA	VIDA Insights	2019	Yes	Product name was LungPrint® Discovery
Visage Imaging, Inc.	Visage Breast Density	2020	No	
Visiana	BoneXpert	2009	Yes	
Visionairy Health	X1	2021	No	
Viz.ai	Viz LVO	2018	Yes	
Volpara Health	VolparaDensity	2010	Yes	Vendor name was Volpara Solutions
VUNO	VUNO Med®-BoneAge™	2018	Yes	
	VUNO Med®-Chest X-ray™	2020	No	
	VUNO Med®-DeepBrain™	2020	No	
	VUNO Med®-LungCT AI™	2020	No	

Table S2. Overview of the 173 CE-marked products included in the 2023 analysis, detailing vendor name, product name, year of market introduction, whether the product was analysed in both the 2020 and 2023 analyses, and any changes to product or vendor names since 2020.

	2020	2023	% Difference (95% CI)	*p*-value*
Total studies	237 (100%)	421 (100%)	N/A	N/A
Level 1	54 (22.8%)	128 (30.4%)	7.62 [0.70, 14.53]	0.04
Level 2	153 (64.6%)	241 (57.2%)	-7.31 [-15.02, 0.40]	0.08
Level 3-6	53 (22.4%)	99 (23.5%)	1.15 [-5.52, 7.83]	0.81
Level 3	*37 (15.6%)*	*67 (15.9%)*	*0.30 [-5.49, 6.10]*	*1*
Level 4	*14 (5.9%)*	*28 (6.7%)*	*0.74 [-3.09, 4.57]*	*0.83*
Level 5	*9 (3.8%)*	*10 (2.4%)*	*-1.42 [-4.26, 1.41]*	*0.42*
Level 6	*4 (1.7%)*	*9 (2.1%)*	*0.45 [-1.69, 2.59]*	*0.78*

Table S3. Levels of efficacy assessed by peer-reviewed papers included in the 2020 and 2023 analyses. A single paper may address multiple levels. N/A = not applicable. **p*-values are based on the chi-square test or Fisher's exact test.

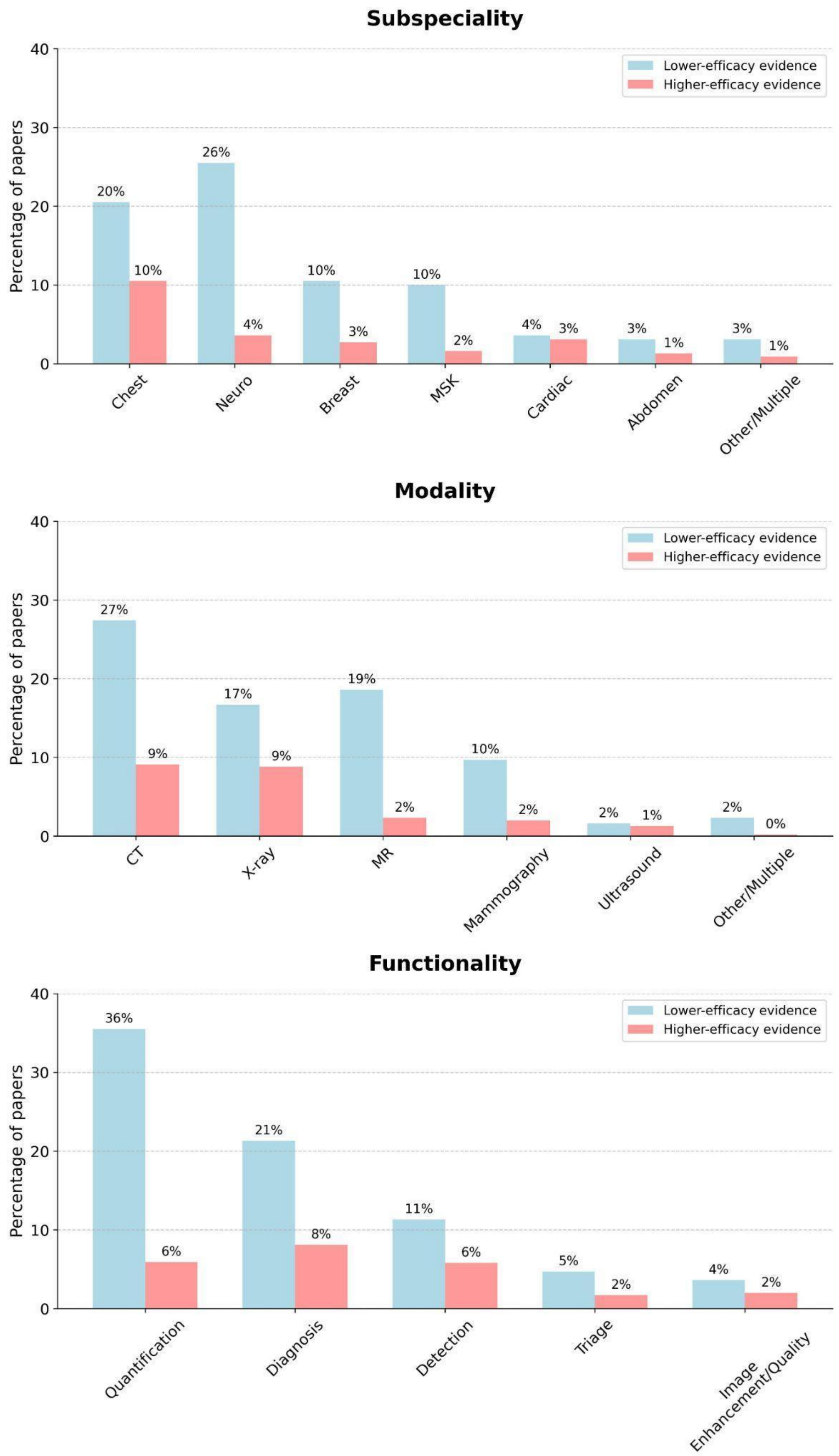

Figure S1. Distribution of peer-reviewed evidence across radiological subspecialities, modalities, and functionalities, as described in Figure 2. Grouped bar charts display the percentage of included papers (n = 639) associated with their respective product categories. Results are stratified by evidence level, with lower-efficacy evidence (levels 1–2) and higher-efficacy evidence (levels 3–6) shown separately. MSK = musculoskeletal.

Eur Radiol (2025) Eur Radiol (2025) Antonissen N, Tryfonos O, Houben IB, Jacobs C, de Rooij M, van Leeuwen KG.

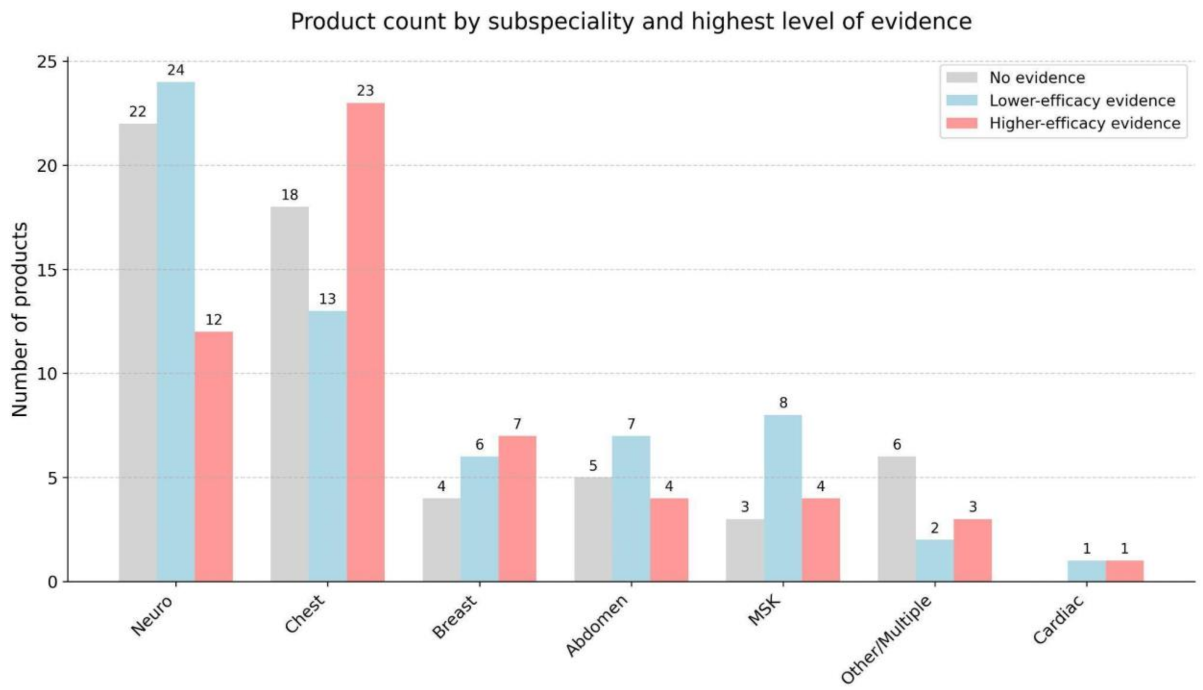

Figure S2. Number of CE-marked AI products in radiology categorised by organ-based subspeciality and their highest level of supporting peer-reviewed evidence. Products are grouped into three categories: no evidence, lower-efficacy evidence (levels 1–2), and higher-efficacy evidence (levels 3–6). MSK = musculoskeletal.
